# Stereotactic partial breast irradiation in primary breast cancer: A comprehensive review of the current status and future directions

**DOI:** 10.3389/fonc.2022.953810

**Published:** 2022-10-13

**Authors:** Silvia Takanen, Paola Pinnarò, Ilaria Farina, Francesca Sperati, Claudio Botti, Patrizia Vici, Antonella Soriani, Laura Marucci, Giuseppe Sanguineti

**Affiliations:** ^1^ Radiation Oncology, Istituto di Ricovero e Cura a Carattere Scientifico (IRCCS) Regina Elena National Cancer Institute, Rome, Italy; ^2^ Biostatistics, Istituto di Ricovero e Cura a Carattere Scientifico (IRCCS) Regina Elena National Cancer Institute, Rome, Italy; ^3^ Surgery, Istituto di Ricovero e Cura a Carattere Scientifico (IRCCS) Regina Elena National Cancer Institute, Rome, Italy; ^4^ Phase IV Studies, Istituto di Ricovero e Cura a Carattere Scientifico (IRCCS) Regina Elena National Cancer Institute, Rome, Italy; ^5^ Physics, Istituto di Ricovero e Cura a Carattere Scientifico (IRCCS) Regina Elena National Cancer Institute, Rome, Italy

**Keywords:** breast cancer, stereotactic partial breast radiotherapy, local toxicity, SBRT, breast cosmesis, APBI, SPI

## Abstract

In selected low-risk breast cancer patients, accelerated partial breast irradiation (APBI) may represent an alternative option to the whole breast irradiation to reduce the volume of irradiated breast and total treatment duration. In the last few years, preliminary data from clinical trials showed that stereotactic partial breast radiotherapy may have the advantage to be less invasive compared to other APBI techniques, with preliminary good results in terms of local toxicity and cosmesis: the use of magnetic resonance, fiducial markers in the tumor bed, and new breast devices support both a precise definition of the target and radiation planning.

**Systematic review registration:**

https://www.crd.york.ac.uk/prospero/display_record.php?ID=CRD42021257856, identifier CRD42021257856.

## Introduction

During the novel coronavirus disease 2019 (COVID-19) pandemic, cancer centers considered shortened courses of radiotherapy (RT) to minimize the risk of infectious exposure of patients and staff members improving the use of new treatment approaches, such as stereotactic body RT (SBRT).

Over the last few years SBRT, combining high radiation doses per fraction with precision targeting, is increasingly used to treat a large variety of localized primary tumors, including non-small cell lung cancer (NSCLC), hepatocellular carcinoma, and pancreatic and prostate cancers (1–8).

In primary breast cancer (BC), the use of stereotactic techniques is still under investigation and its interest is growing in the context of accelerated partial breast irradiation (APBI) (9, 10). Considering that most of local recurrences (LRs) are at the level of the same quadrant of the original tumor (10, 11), APBI, in early BC patients, can offer an alternative to the standard whole-breast RT (WBRT) after breast-conserving surgery (BCS). The appropriate candidates for APBI are selected according to international guidelines (12).

In most important randomized clinical trials, different technical approaches have been evaluated for APBI: external beam irradiation (EBRT) (13, 14) and brachytherapy (15–18), which commonly use 10 fractions over 1 week, or intraoperative electron RT (IOERT) (19, 20).

Compared to the previous cited techniques, stereotactic PBI (SPBI) may have the advantages of being less invasive and faster due to focusing on the target along with the use of a higher dose of radiation per fraction and the reduction of the dose to the surrounding normal tissues. Moreover, it improves the accuracy of treatment through the different currently available machine devices, such as real-time tracking, prone position, breast devices, or a gating system in order to minimize respiratory motion effects.

Feasibility phase I and II clinical studies in the neoadjuvant and adjuvant setting for SPBI have been published, and there are actively enrolling trials.

This paper is a comprehensive review aimed to examine the current evidence focused on the use of SBRT for primary early BC to assess the potential advantages of this technique in the context of APBI.

## Materials and methods

**Figure 1:**
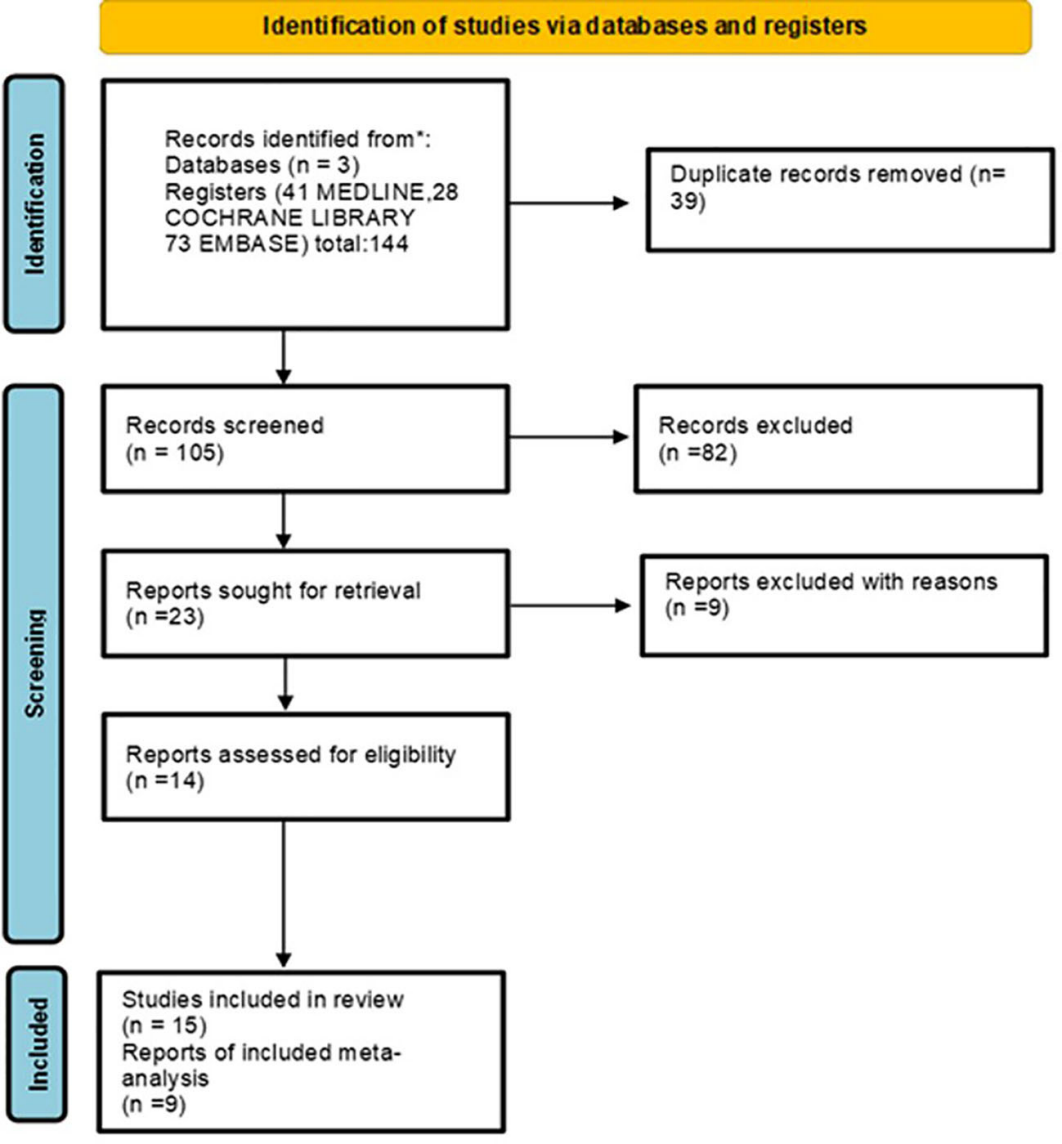
Flow diagram of the process of study identification and selection.

A comprehensive search strategy is provided in **Figure 1**. Five databases were searched: Pubmed, Embase, and Cochrane databases with a date range from 2005 to 2022. Search terms were formulated using the PICO structure. Participants (P) included women affected by early BC undergoing BCS. Intervention (I): SPBI. Comparisons (C): preoperative versus postoperative SPBI. Outcomes (O): LC, local toxicity and cosmesis. Using the following MESH terms (((“Breast Neoplasms”[Mesh]) OR ((Breast[Title/Abstract] OR Mammary[Title/Abstract]) AND (Neoplasm*[Title/Abstract] OR Tumor*[Title/Abstract] OR Tumour*[Title/Abstract] OR Cancer*[Title/Abstract] OR Carcinoma*[Title/Abstract] OR Adenocarcinoma*[Title/Abstract] OR Malignant[Title/Abstract]))) AND ((“stereotactic body radiation therapy”[Title/Abstract] OR “Stereotaxic Techni*”[Title/Abstract] OR “Stereotactic Techni*”[Title/Abstract] OR “Techni* Stereotaxic”[Title/Abstract] OR “Techni* Stereotactic”[Title/Abstract]) OR (“Stereotaxic Techniques”[Mesh]))) AND ((“Mastectomy, Segmental/methods”[Mesh]) OR (“partial breast irradiation”[Title/Abstract] OR pbi[Title/Abstract] OR apbi[Title/Abstract])).

The protocol was published in the PROSPERO international prospective register of systematic reviews with the following registration number: CRD42021257856.

### Study identification and selection

The search was filtered for English or French language and clinical trial. After removing duplicates, we screened the titles and abstracts of 144 records on 30th March 2022 after we applied the filter “clinical trial” with a total of 12, 28, and 74 Pubmed, Cochrane, and Embase articles, respectively. We excluded 119 records, selecting 23 for full-text reading.

Finally, 14 records were excluded due to the lack of accrual and no further funding.

We therefore included 15 records reporting the results. We used Cochrane guidelines for systematic reviews (21), and two reviewers (ST and IF) independently assessed the quality of each study.

### Inclusion and exclusion criteria

We included only prospective and retrospective patient cohorts or randomized studies reporting on clinical outcomes (LRs and toxicity) in the setting of SPBI as they are considered to be the best type of scientific studies to answer questions about the potential advantages of the use of SPBI. Reports regarding the use of SPBI only boost at the level of the tumor bed; the use of SBRT in oligometastatic BC was excluded. Different stereotactic techniques were also included. Case reports, abstracts, and review papers were excluded. The reference lists of selected publications were further searched for relevant articles. Abstracts were reviewed, and a search for the full publication was performed in case the abstract was found to be relevant to the topic, which included attempts to contact the authors for further details if the contact information was available.

The flow diagram of the process of study identification and selection is presented in **Figure 1**.

### Data extraction and statistical analysis

Data were analyzed based on the following themes: LR rate (including in-breast relapse, in the same quadrant or in quadrants other than the primary, and locoregional nodal relapse), risk factors for LRs (e.g., tumor stage, histology, grade, resection margin status, and skin or lymph node involvement), and toxicity outcomes (local acute toxicity). Summary statistics were calculated using the crude rates of events pooled.

Only adjuvant studies included all selected endpoints.

The toxicity and cosmesis event rate were pooled to determine an overall summary estimate for each outcome. We reported forest plots, assessing their corresponding 95% confidence interval (CI) and the angular transformation, and a 0.5 continuity correction for studies with an event proportion of 0 or 1 was applied. Heterogeneity among studies was assessed by a visual inspection of forest plots, an estimation of the percentage heterogeneity between studies that could not be ascribed to sampling variation (I^2^ test) (22), and a formal statistical test of the significance (23). An I^2^ value equal to or lower than 25% as trivial heterogeneity and an I^2^ of 75% or higher were considered as a factor of important heterogeneity.

## Results

A final list of 15 articles (24–38) (**Tables 1**, **2**) were included in full manuscript review. All studies included patients with early BC who were treated over 8 years from 2009 and 2021.

**Table 1:** Neoadjuvant phase I-II clinical trials on SPBI.

STUDY	STUDY TYPE	PATIENTS (n)	TREATMENT PRESCRIPTION	MAIN FINDINGS
Palta et al. (24)	Prospective	17	Total dose 15 Gy in one fraction	Dosimetric feasibility in tumors >1 cm from the skin Median percentage of the ipsilateral breast volume receiving 100% and 50% of the prescribed dose in preoperative SPBI significantly lower than in postoperative control group (p= 0.002)
Blitzbau et al. (25)	Prospective	32	Three single-fraction cohorts (15,18, 21 Gy)	MR as fundamental support for target delineation Target volumes markedly smaller than historical postoperative volumes Negligible doses to normal surrounding tissues
Horton et al. (26)			Prospective		32	Three single-fraction cohorts (15,18, and 21 Gy)	Main outcomes at 2 years comparable to standard WBI Analysis of gene expression as radiation response biomarkers and therapeutic targets to enhance radiation sensitivity in more resistant tumors
Wang et al. (27)	Prospective	15	Three single-fraction cohorts (15,18, and 21 Gy)	MR quantitative parameters as radiation response biomarkers
Guidolin et al. (28)			Prospective	52 accrued 27 treated	Total dose 21 Gy in one fraction	No grade ≥2 toxicity at 3 weeks or 1 year postoperatively 1 pt grade 2 delayed wound infection at 6-month postoperative visit MR as fundamental support for target delineation
Weinfurtner et al. (29)	Prospective	20			Total dose 28.5 Gy in three fractions	Strong linear correlation of MR %VC to %TC High accuracy of %VR on MR to predict pPR in ER/PR+ and Her2- patients

**Table 2:** Adjuvant phase I–II clinical trials on SPBI.

STUDY	STUDY TYPE	PATIENTS (n)	TREATMENT PRESCRIPTION	MEDIAN FOLLOW UP (MONTHS)	CLINICAL OUTCOMES	LOCAL CONTROL
Vermeulen et al. (30)	Retrospective	46	Total dose 25–36 Gy in 5–10 fractions	31 (21 pts) 21 (26 pts)	3 pts dermatological toxicity, 1pt lumpectomy pain Good/excellent cosmesis	LRFS 100%
Obayomi et al. (31)	Retrospective	10	Total dose 25–36 Gy in 5–10 fractions	15	1 pt dermatological tox (fibrosis) Good/excellent cosmesis	LRFS 100%
Lozza 2018 (32)	Prospective	29	Total dose 30 Gy in 5 fractions	27.7	8 pts skin erythema 6 pts skin dry and edema 17 pts fibrosis 10 pts hyperpigmentation 1 pts skin ulceration and atrophy Good cosmesis	LRFS 100%
Rahimi et al. (33)	Prospective	75	Five cohorts of 15 patients Five-fraction dose escalation (2.5 Gy) from 30 Gy in the first cohort	61	11 pts fat necrosis 5 pts breast pain Cosmesis non evaluated	LRFS 100%
Rahimi et al. (34)	Prospective	75	Five cohorts of 15 patients Five-fraction dose escalation (2.5 Gy) from 30 Gy in the first cohort	60	71 pts Good/excellent cosmesis at 5 yrs	LRFS 100%
Rahimi et al. (35)	Prospective	30	Single fraction Three cohorts of 29 patients (11 patients treated dose 22.5 Gy; 8 patients treated dose 26.5 Gy; 10 patients treated dose 30 Gy	24.7	1 pts skin dermatitis 13 pts fat necrosis 3 pts breast pain 1 pts fair cosmesis	LRFS 100%
Mészàros et al. (36)	Prospective	27	Total dose 25 Gy in four fractions	12	6 pts skin erythema 3 pts edema 2 pts breast pain Good/excellent cosmesis	LRFS 100%
Lee et al. (37)	Retrospective	104	Total dose 30 Gy in five fractions	3	4 pts skin induration (fibrosis) Cosmesis not evaluated	LRFS 100%
Cervide et al. (38)	Retrospective	23	Total dose 30 Gy in five fractions	66	1 pts skin dermatitis Good/excellent cosmesis	LRFS 100%

### Neoadjuvant trials

The rationale of neoadjuvant SPBI is to irradiate smaller treatment volumes in an attempt to improve cosmetic outcomes and minimize the risk of the late effects of the surrounding breast tissue.

We found six published trials (**Table 1**) related to the use of SPBI neoadjuvant to surgery.

Palta et al. published in 2012 a 15-Gy single-fraction preoperative SPBI pilot study in 17 patients affected by early BC (24). They designed a phase I trial to dosimetrically analyze potential differences between pre- and postoperative PBI, and they found a dose reduction in ipsilateral breast tissue in the preoperative SPBI group compared to institutional postoperative PBI historical controls. The median percentage of the ipsilateral breast volume receiving 100% and 50% of the prescribed dose in preoperative SPBI was 3.8% and 13.3%, respectively, compared with 18% and 53%, respectively, in the institutional historical controls treated with postoperative external beam PBI (p = 0.002). Absolute doses to the heart and ipsilateral lung were negligible. In their study, they also considered the impact of the dose to the skin that had been shown to be predictive of long-term side effects in SBRT (Hoppe et al., 2008). To this end, the clinical target volume (CTV) was created as a uniform 1.5-cm expansion around the gross tumor volume (GTV). The first 5 mm of subcutaneous tissue and any chest wall structure (pectoralis muscle and deeper) >1 cm from the GTV was excluded from the CTV expansion. The first 5 mm of subcutaneous tissue is in the photon ‘‘build-up’’ region, and including this area would skew the dose calculations. The median skin maximum dose (D_max_) and the D_max_ to 1 cm^3^ of the skin were 9 and 6 Gy, respectively. They concluded that preoperative, single-fraction SPBI is dosimetrically feasible in women with small tumors at least 1 cm away from the skin; the skin dose appears reasonable given the small volumes, although additional studies are required to determine whether these doses are tolerable in terms of short- and long-term toxicity.

From these preliminary results, the Duke University group developed a prospective phase I trial (25) to evaluate the feasibility of a preoperative single-fraction SPBI. A total of 32 patients were treated in a prone position with three single-fraction cohorts (15, 18, or 21 Gy). Magnetic resonance (MR) was used for target delineation, and a prone position setup was reproduced; they easily met normal tissue constraints from PBI and lung SBRT protocols for all patients, likely due to the smaller target volumes than postoperative PBI ones. Therefore, they concluded that it would be possible to define stricter normal tissue constraints to minimize potential acute and chronic toxicity rates with the preoperative approach. Regarding the skin dose, they achieved a mean skin D_max_ of 14 +/− 3 Gy and the doses to 1 and 10 cc of skin were 11 +/− 3 and 7 +/− 2 Gy, respectively. The CTV and planning target volume (PTV) expansions required modifications to meet the trial-mandated avoidance of the first 5 mm of subcutaneous tissue for 16 patients. However, the resulting mean percent volume change was only 4% for CTV and 6% for PTV. They concluded that it is not necessary to trim the PTV near the skin surface given the lack of toxicity observed in their study.

In a subsequent preoperative phase I SPBI trial with the same three dose-level single fraction cohorts (26), at a median follow-up (fup) of 23 months, there has been no LRs. Cosmetic outcomes were good/excellent, and no severe or higher (grade 3+) late toxicities were reported. Pre- and postradiation MR images and patient tumor samples were analyzed, showing a linear correlation between the treatment dose and both vascular permeability and cell density. The modifications of gene expression profiles were found after radiation. The impact of radiation on relative gene expression increases with each incremental increase in dose, and the primary effect of dose escalation is to enhance, rather than repress, gene expression, experiencing significant and dose-related change with radiation. The cohort of analyzed genes demonstrating significant dose–response is enriched for the modulators of immunity and inflammation, and they could represent a path forward to identify radiation response biomarkers, particularly in more radio-resistant tumors.

Preoperative PBI also offered the opportunity to evaluate differences at MR in pre- and postradiation imaging in order to better understand radiation response and potentially identify the functional imaging radiological biomarkers that can be used as prognostic and predictive tools. Wang et al. (27) developed a novel clinical trial evaluating the use of a highly conformal preoperative SBRT approach with the single-fraction delivery using dynamic contrast-enhanced magnetic resonance imaging (DCE-MRI) and diffusion-weighted magnetic resonance imaging (DW-MRI) for radiation response investigation. The primary objective of the study was to assess the relative changes in selected parameters after radiation treatment; the secondary objective was to investigate the potential linear relationship between parameter changes and the delivered radiation dose. They confirmed that MR quantitative parameters (both DW and DCE imaging) could potentially be used as radiation response biomarkers. A linear relationship between the RT dose and the relative parameter changes was observed for various MR quantitative parameters.

The SIGNAL single-arm trial (28) showed, in 27 early BC patients treated in the prone position, the safety and feasibility of a 21 Gy single dose of preoperative SPBI. Although acute toxicity and cosmesis were acceptable, they did not comment about late radiation toxicity due to the short fup. They confirmed the importance of MR to delineate the target volume and the prone setup to minimize the effect of respiratory motion. They include a minimum distance of each lesion of at least 2 cm from the skin. The unique aspect of the SIGNAL trial includes the use of volumetric modulated arc therapy (VMAT) along with strict dosimetry constraints to the skin and normal tissues. Consistently, the authors did not observe any early skin erythema or dermatitis, as well as any grade of fibrosis or telangiectasia by the latest fup at 1 year.

The MR response to preoperative SBRT was evaluated in another recent single institution phase II trial (29): 19 early ER/PR+ and Her2 - BC patients underwent SBRT in three fractions (total dose 28.5Gy), followed by an MR fup from 5 to 6 weeks after SBRT. The tumor size and BI-RADS descriptors on pre- and post-SBRT breast MRs were compared to evaluate the correlation with the percentage of tumor cellularity (%TC) at surgical findings. Reported MR tumor sizes were used to calculate the percent cubic volume remaining (%VR). Four patients had MR CR, but no patient achieved pCR. MR %VR demonstrated a strong linear correlation with the percentage of tumor cellularity (%TC) (p =.0008) and high accuracy (89%) for predicting partial pathologic response (pPR) (sensitivity 88%, specificity 100%). The authors conclude that tumor %VC assessed on MR after neoadjuvant SBRT could permit to identify ER/PR+ and Her2- patients, rarely achieving a pCR, who can benefit from preoperative RT.

### Adjuvant trials

We found nine trials evaluating SPBI in an adjuvant setting (**Table 2**).

In a small study (30), 47 patients were treated with SPBI performed in 5–fractions by tracking and correcting for respiratory motion with the Synchrony respiratory motion management system of CyberKnife (CK): after a median fup of 22 months, no serious toxicities (grade ≥ 2) were observed with an excellent cosmetic result in all treated patients.

In 2016, the Georgetown University (31), in a small group of patients with early BC, explored the feasibility of SPBI delivered in five fractions to the total dose of 30 Gy. A synchrony system tracked the intrafraction motion of four gold fiducials implanted around the lumpectomy cavity prior to treatment under ultrasound guidance. At a median fup of 1.3 years, all patients experienced excellent/good breast cosmesis outcomes without any breast recurrence.

Preliminary results from another 2-year pilot study by Lozza et al. (32) showed good cosmetic outcomes with mild acute and late toxicity rates. During surgical intervention, after the removal of the breast tumor, 2 mm gold seeds used as fiducial markers for driving CK-PBI were positioned around the surgical cavity after the breast glandular reshape and before the suture of the surgical wound. These gold seeds were conventionally positioned on three different spatial planes, at the three corners of an ideal triangle centered on the area in which the tumor was removed. All 29 patients received the prescribed dose of 30 Gy in five fractions to the tumor bed PTV. After a median 24-month fup, there were no recurrences. They confirmed that cosmesis is dependent on the target volume size and consequently on the volume of the ipsilateral breast treated: with a prescribed isodose line of 87%, V50 and V100 levels on average were kept to 28.7% and 10.7%, respectively. No patient experienced a fair/poor cosmesis or fibrosis, and good/excellent cosmetic rates were recorded. Indeed, CK allows a relatively steep dose gradient just outside the target volume.

Rahimi et al. (39) confirmed the satisfactory results in a dose-escalation phase I to 40 Gy with only one event of dose-limiting toxicity (grade 3 dermatitis). In 2020, an update of this study was published (33): fat necrosis was evaluated in 75 early BC patients. After a median fup of 61 months, 11 patients experienced palpable fat necrosis, five cases of which were painful. The predictive factors for fat painful necrosis resulted to be higher V35 and -50 Gy (p <.05), and the administration of two treatments on consecutive days (p= 0.02). They concluded that SPBI shows a fat necrosis rate comparable to other PBI techniques but it is less invasive. However, to reduce the risk of painful fat necrosis, the authors suggest not to deliver the radiation treatment on consecutive days and to consider caution when radiating women with larger than 1,063 cm^3^ breast size. Fat necrosis dose constraints were also proposed and being tested in an additional Phase II SPBI fraction trial (40).

The first results regarding cosmesis evaluated through the Harvard Breast Cosmesis Scale were published in 2021 (34): most patients reported excellent/good cosmesis at both baseline (86.3%) and year 3 (89.8%), and no cosmetic subdomain (telangiectasia, skin atrophy, scarring, pigment change, erythema, fat necrosis, and fibrosis) had significant worsening by year 3.

The same American team (35) recently published preliminary results on the toxicity and cosmesis of a multicenter trial using single-fraction postoperative SPBI. A total of 30 patients were evaluated with a median fup of 24.7 months; three dose levels were used (22.5, 26.5, or 30 Gy) in a single fraction at the level of the tumor bed delivered through the Synchrony respiratory tracking system of CK. The target was localized by four fiducial markers or the BioZorb system (41), which is an open spiral device that incorporates six permanent titanium clips in a fixed three-dimensional (3D) array and provides landmarks at the site of a tumor after it has been surgically removed. No dose cohort had a statistically significant cosmetic detriment. No patient developed grade 3 dermatitis; two patients developed grade 2 breast pain, and one patient developed grade 3 breast pain: on univariate logistic regression, no dosimetric parameters were found to significantly increase the risk of developing grade 2 breast pain. Four of the 29 treated patients developed fat necrosis: two patients in both the 22.5 and 26.5 Gy cohorts developed fat necrosis, and none in the 30 Gy cohort and those who developed fat necrosis were found to have significantly larger PTVs using fewer beams. Furthermore, they evidenced that 42.8% of patients who had Biozorb placed at the time of surgery developed fat necrosis. These results compare favorably to other large studies evaluating the risk of fat necrosis after both WBI and PBI. They also showed promising cosmetic outcomes with single-fraction SPBI, with no significant cosmetic detriment by month 12 in the highest dose cohort.

The Hungarian team (36) reported preliminary results on 27 patients with early BC treated in the context of a phase II trial (median fup less than 1 year): In this study, SPBI delivered a total dose of 25 Gy in four fractions and CK was used. No grade >2 acute toxicities were observed as well as no LRs or distant metastasis. Cosmetic results were good and excellent for all patients.

The Korean team (37) reported their first experience on the use of SPBI in 104 early-BC patients, finding that it is dosimetrically and technically feasible and clinically safe for selected low-risk patients: after a median fup of 13 months, no immediate post-SPBI toxicity ≥ grade 2 was reported, except grade 2 induration in three breasts. All patients remain disease-free to date.

A recent phase II trial (38) was published with the longest fup, assessing 23 early-BC patients for the feasibility of postoperative SPBI and the ExacTrac Adaptive Gating System: with a median fup of 66 months, LR-free survival reaches 100%. One patient developed a second BC outside the treated quadrant after 25.1 months.

**Figure 2:**
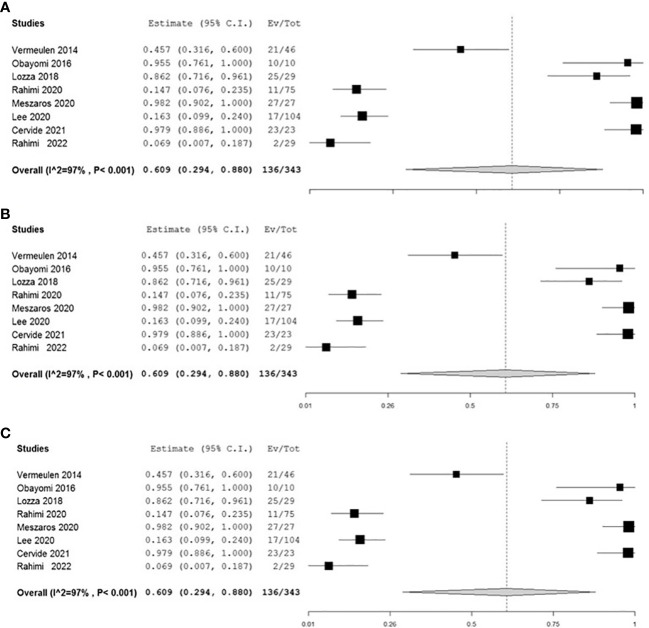
Forest plots showing the high heterogeneity of adjuvant studies: the first forest plot **(A)** concerns the acute local toxicity evaluated in 8 studies; the other two forest plots **(B, C)** included all 9 studies evaluating local cosmesis.

We pooled the overall acute toxicity (**Figure 2A**) and the excellent/good cosmetic event (**Figures 2B, C**) rate to determine an overall summary estimate for each outcome.

A high statistical heterogeneity (p<0.001) has been evidenced among all adjuvant studies for both outcomes (**Figures 2A–C**): this result could be explained by the differences, among all adjuvant studies, related to the study design’s characteristics (e.g., number of patients and length of fup), that make it difficult to develop a consistent metanalysis.

## Discussion

During the challenging time of the COVID-19 outbreak, the use of APBI in early BC, with the advantage of reducing the total treatment of time and the number of accesses to the hospital, could be a valid solution to the emergency, especially the one–three fraction schedules. At the same time, it offers the possibility to cope with general problems concerning the distance from RT centers and other difficulties, such as comorbidities, to attend a daily treatment, with the consequent omission of adjuvant RT.

To our knowledge, this is one of the first comprehensive and systematic reviews specifically addressing the topic of the use of SPBI in primary early low-risk BC in either the neoadjuvant or the adjuvant settings (42).

Concerning the main objective of this review, we can conclude that SPBI in early BC represents an increasingly attractive option for the possibility of using shorter fractionation schedules, focusing on the target, and the non-invasive characteristic of the technique compared to other APBI treatments (20, 43–45).

Although the limitation of preoperative SPBI studies (short fup and a very small sample of patients with focus on dosimetric, biological, and radiological analysis), neoadjuvant stereotactic treatments, compared to adjuvant SPBI, permits a more precise distribution of the dose at the visualized target, allowing a dose de-escalation up to a single-fraction treatment (28) and reducing the radiation dose to the normal surrounding tissues. The disadvantage of preoperative SPBI seems to be similar to the IORT technique: after BCS, if pathology reveals tumor characteristics outside entry criteria (e.g., large tumor size and nodal involvement), WB and regional nodal (as indicated) RT must be delivered (25).

Currently, the use of gold fiducials at the level of tumor bed and the evolution of radiation techniques, such as MR-guided RT (26–28), the real-time tracking and respiratory motion control of the CK system (46) or ExacTrac Adaptive Gating System (Novalis^®^) (38, 47, 48), and some new breast devices (49–51), have also heightened the interest in the use of SPBI in the adjuvant setting, reducing radiation delivery uncertainty related to the pliability and mobility of the breast tissue and a more accurate dose distribution, limiting the dose at OARs.

The irradiated volume and breast normal tissue around the target showed to represent an important factor to predict local toxicity (24, 28, 31–33). MR-guided systems (52) offer an effective guide for each step of RT, from simulation to contouring to treatment planning and delivery to treatment response evaluation. The main advantage is the reduction or the omission of CTV to PTV margins both in neoadjuvant and adjuvant PBI with a better visualization of the target volume during treatment. PTV intrafraction motion can be controlled through the irradiation delivered in a selected gated area of the respiratory cycle with respiratory gating systems (38). The modern CK radiosurgery system also delivers a relatively steep dose gradient outside and within the target, mimicking the accurate brachytherapy dose distribution, and it proved to limit normal breast tissue toxicity with good cosmesis (30–32, 35, 37, 46, 53).

We must underline the fact that only preliminary results in terms of the toxicity extracted by phase I and II trials with small sample of patients are available. Moreover, the data regarding late toxicity and LC are limited due to the short median fup.

However, in the last several years, the interest for this SBRT in the context of PBI in low-risk BC is growing and the number of ongoing trials evaluating the role of SPBI in a neoadjuvant (54–58) and adjuvant setting (59–63) (**Table 3**) are increasing.

**Table 3:** Ongoing trials on neoadjuvant and adjuvant stereotactic partial breast irradiation.

STUDY	STUDY TYPE	TREATMENT PRESCRIPTION	END POINTS
NCT03581136 (40)	Prospective trial	30 Gy in five fractions	Quality of life Cosmesis
NCT02316561 (54)	Prospective trial	Single fraction of 15 Gy	cPR radiological response toxicity
NCT02065960 (55)	Prospective trial	40 Gy/five fractions every other day over a period of 10–12 days	Feasibility, toxicity and LC
NCT02482376 (56)	Prospective trial	Single fraction of 21 Gy	Cosmesis
NCT04807192 (57)	Prospective trial	Single fraction of 8 Gy alone (arm1) or with IT CMP001 (arm2)	cPR and pPR LC DFS OS tumor biological activity
NCT04040569 (58)	Prospective trial	Single-fraction SBRT cohorts	MTD Cosmesis LC
NCT03043794 (59)	Prospective trial	Single fraction of 21 Gy	cPR pPR cosmesis quality of life LC
NCT04234386 (60)	Prospective trial	Single-fraction SBRT cohorts	MTD DLTs LC
NCT 02457117 (61)	Prospective trial	30 Gy in five fractions over 5–14 days	Local failure and patterns of in-breast failure
NCT 02685332 (62)	Prospective trial	Single-fraction SBRT cohorts	MTD Dose-limiting toxicity Cosmesis LC
NCT03568981 (63)	Observational trial	PBI or WBI after surgery according to the international guidelines and physician’s discretion	Cosmetic outcome and breast tissue fibrosis in early BC

The ABLATIVE single-arm prospective trial (54) from the Netherlands, in the context of APBI, has the pCR as a primary endpoint after a single dose of SBRT by using contrast-enhanced and functional MR scan for planning. A total of 25 consecutive patients will be treated with a single ablative RT dose of 20 and 15 Gy to the tumor bed. The secondary study endpoints are the radiological response and toxicity.

In Canada, the feasibility and, as a secondary endpoint, the toxicity and pathological outcomes are evaluated in women aged 70 and older in the ARTEMIS trial (55). SBRT to a dose of 40 Gy in five fractions was delivered every other day over a period of 10–12 days, followed by BCS.

Another American ongoing phase II trial (56) is exploring the results of SPBI in a preoperative setting to evaluate the rate of excellent/good cosmesis, the pathological response, and the impact of radiation on gene expression. The first results presented at the ASTRO 2020 congress (64) showed that preoperative SPBI failed to achieve pCR at 6–8 weeks following treatment, although it was well tolerated and improved convenience for most patients. Eligible patients underwent preoperative SBRT to 28.5 Gy in three fractions of 9.5 Gy/fraction followed by BCS and sentinel lymph node (SLN) evaluation 6–8 weeks later. With a median fup of 13.5 (4–24) months, no LRs have been observed. Four patients were incidentally node-positive following SLN evaluation, two micrometastases and two macrometastases, and underwent post-SBRT WBRT. In the patients who only had SBRT, 15% (3) and 5% (1) developed late grade 2 or 3 soft-tissue toxicity, respectively. The conclusion was that the resultant addition of WBRT in these cases may have increased the risk of grade 3 late toxicity.

The adjuvant SPBI trials include a five-fraction prospective registry trial (60, 61), and a 30 Gy in a five-fraction phase I–II adjuvant partial breast trial (39, 63).

The University of Texas Southwestern is also conducting a single fraction phase I adjuvant dose-escalation SPBI trial with the starting dose of 22.5 Gy to establish the maximum dose tolerated and the dose-limiting toxicity for each dose level (62). The same team is following a neoadjuvant phase I trial (58) to evaluate dose-limiting toxicity while dose-escalating single-fraction preoperative SPBI to a presumed radioablative dose over three cohorts, from 30 Gy in one fraction to 34 and 38 Gy in one fraction.

In 2018, the University of South Korea started a prospective trial selecting early BC patients eligible to postoperative WBRT or SPBI, delivered in five fractions, to assess patient-reported outcomes, the cosmetic outcome, and breast tissue fibrosis (63).

Considering the growing importance of this treatment option (65) in other pathologies, we await with great interest the results of the ongoing trials to define the role of this new technique in the context of early BC.

## Conclusion

Notwithstanding the limitations of the analyzed studies, the available data here discussed for the use of SPBI allow us to conclude that this approach is feasible and its interest is growing: the potential advantage of the stereotactic technique is that, compared with other APBI techniques, it is less invasive, reducing the irradiated breast tissue surrounding the target volume. The use of the MR-guided technique can facilitate the target delineability, patient positioning, and intrafraction motion monitoring and may represent support to predict and assess the radiation treatment response.

In conclusion, it is not time for prime time.

## Data availability statement

The original contributions presented in the study are included in the article/supplementary material. Further inquiries can be directed to the corresponding authors.

## Author contributions

ST contributed to the conception and design of the work and wrote the first draft of the manuscript. FS developed statistical analysis. IF conceived the methodological approach. PP reviewed the final version. All authors contributed to the last manuscript revision and approved the submitted version.

## Acknowledgments

This work was supported by Funds Ricerca Corrente 2022 from Italian Ministry of Health. The use of research databases was supported by Dr. Francesca Servoli, Digital Library “R. Maceratini” IRCCS, Istituto di Ricovero e Cura a Carattere Scientifico Regina Elena National Cancer Institute of Rome (Italy).

## Conflict of interest

The authors declare that the research was conducted in the absence of any commercial or financial relationships that could be construed as a potential conflict of interest.

## Publisher’s note

All claims expressed in this article are solely those of the authors and do not necessarily represent those of their affiliated organizations, or those of the publisher, the editors and the reviewers. Any product that may be evaluated in this article, or claim that may be made by its manufacturer, is not guaranteed or endorsed by the publisher.
